# Supplementary material for: Modelling how cleaner fish approach an ephemeral reward task demonstrates a role for ecologically tuned chunking in the evolution of advanced cognition
Source: PLoS Biol. 2022 Jan 5;20(1):e3001519. doi: 10.1371/journal.pbio.3001519 (PMC8765642; doi:10.1371/journal.pbio.3001519)
Supplement: S1 Text — (PDF) [file pbio.3001519.s003.pdf]

Modelling how cleaner fish approach an ephemeral reward task demonstrates a role for ecologically-tuned chunking in the evolution of advanced cognition

Yosef Prat[1*], Redouan Bshary[1], Arnon Lotem[2*]

[1] Institute of Biology, University of Neuchâtel, Switzerland.

[2] School of Zoology, Faculty of Life Sciences and Sagol school of Neuroscience, Tel Aviv University, Tel Aviv, Israel.

* Corresponding authors: yosefprat@gmail.com, lotem@tauex.tau.ac.il

Supporting Information

Laboratory complex market problem

Following the experiments conducted by Truskanov et al. [1]. we simulated another environment of the market problem: the *lab complex market problem*. In this environment the cleaner faces a visitor-resident combination in 0.5 of the feeding trials, a resident-resident combination in 0.25 of the feeding trials, and a visitor-visitor combination in 0.25 of the feeding trials. As in the standard *laboratory market problem*, each feeding trial is followed by an empty trial. In the *lab complex market problem*, the extended-credit model generates only weak preference towards the visitor (S3 Fig, yellow line), yet it still did better than the core model or the linear operator that choose clients with equal probabilities (S3 Fig, orange and blue lines). The reason for this minor preference is that in the *lab complex market problem* serving a second client after serving a visitor is still more frequent than serving a second client after serving a resident (even before any preference has been developed). This is because serving a visitor is followed by an empty trial only in the case of two visitors being presented (one is served and the other leaves), while serving a resident is followed by an empty trial in all combinations and choices except for a resident followed by another resident (in the visitor-resident choice, choosing a visitor means the resident is served last and choosing a resident also makes it last as the visitor leaves, while in the resident-resident case one is being served first and then the second resident is followed by an empty trial). Thus, the extended-credit model would assign somewhat higher value to the visitor and, subsequently, can develop some preference towards the visitor according to its decision rule.

References

1. Truskanov N, Emery Y, Porta S, Bshary R. Configural learning by cleaner fish in a complex biological market task. Anim Behav. 2021;181: 51–60. doi:https://doi.org/10.1016/j.anbehav.2021.08.023
